# Gastric, Colonic, and Rectal Amyloidosis in the Setting of Familial Mediterranean Fever: A Unique Cause of Intractable Diarrhea

**DOI:** 10.1155/2024/6679725

**Published:** 2024-01-23

**Authors:** Lefika Bathobakae, Nida Ansari, Anas Mahmoud, Shayee Hasan, Ruhin Yuridullah, Sohail Qayyum, Sam Rae

**Affiliations:** ^1^Internal Medicine, St. Joseph's University Medical Center, Paterson, USA; ^2^Gastroenterology and Hepatology, St. Joseph's University Medical Center, Paterson, USA; ^3^Pathology and Lab Medicine, St. Joseph's University Medical Center, Paterson, USA; ^4^Nephrology Division, St. Joseph's University Medical Center, Paterson, USA

## Abstract

Familial Mediterranean fever (FMF) is a hereditary disorder characterized by episodes of fever, polyserositis, or cutaneous inflammation. The FMF attacks last 1–3 days and have no apparent triggers. Recurrent deposition of the serum amyloid A (SAA) protein in the gut can cause intractable diarrhea, dysmotility, and recurrent abdominal pain. Gastrointestinal amyloidosis is a rare, but serious, complication of FMF. In this case report, we describe a rare case of chronic diarrhea and recurrent abdominal pain due to FMF-induced gastrointestinal amyloidosis.

## 1. Introduction

Familial Mediterranean fever (FMF) is an autoinflammatory autosomal disorder that manifests as recurrent episodes of serositis and fever [1, 2] or skin erythema [3]. Reported to have originated in Mesopotamia 3000 years ago, FMF has a higher burden on individuals from the Mediterranean basin [2]. The Mediterranean fever (MEFV) gene, located on the short arm of chromosome 16, encodes the pyrin protein [1]. Pyrin is an innate immune sensor that protects the body against infection and other harmful substances [1, 2]. In FMF, the pyrin inflammasome assembly can be easily triggered by any stimulus, thus forcing the body into a proinflammatory state. The production of inflammatory markers, such as IL-1 and IL-18, results in serositis [1]. Recurrent FMF attacks lead to continued tissue deposition of serum amyloid A (SAA) protein, an acute-phase reactant [4, 5]. SAA protein deposition in the gut causes gastrointestinal (GI) amyloidosis, a rare yet severe complication that often presents as chronic diarrhea and recurrent abdominal pain [4, 6]. Herein, we report a rare case of gastric and colorectal amyloidosis as a unique cause of intractable diarrhea in a patient with a suspected history of adult-onset FMF.

## 2. Case Report

A 48-year-old Syrian man with a medical history of suspected familial Mediterranean fever (FMF), hypertension, end-stage renal disease due to hypertensive nephropathy, and a recent right kidney transplant presented to the emergency department (ED) with generalized weakness and abdominal pain. Abdominal symptoms were described as poorly localized, nonradiating, intermittent, and throbbing pain. These symptoms were accompanied by subjective fever, nausea, vomiting, anorexia, and unquantifiable weight loss. The patient also reported 5-6 episodes of watery diarrhea daily, even with fasting. He denied early satiety, melena, or hematochezia, recent camping, or recent international travel. He also denied any personal or family history of food sensitivity or inflammatory bowel disease. Notably, the patient was seen in the ED multiple times in the preceding six months for the evaluation of vague abdominal pain, intermittent fever, and diffuse joint pain. His daily home medications entailed tacrolimus 4 mg, three times daily, prednisone 5 mg, once daily, and mycophenolate 360 mg, twice daily, for immunosuppression.

In the ED, the patient's vital signs were significant for tachycardia, with a heart rate of 116 beats/minute. The patient was thin-appearing and in distress due to pain. The abdomen was soft, nondistended, and had a healed transplant scar on the right lower quadrant. The abdomen was diffusely tender on palpation, but without guarding, rigidity, or rebound tenderness. Hyperactive bowel sounds were appreciated throughout, and the digital rectal examination was normal. No rashes, palmar erythema, rheumatoid nodules, joint tenderness, or obvious joint deformities were observed. The rest of the physical examination was unremarkable. Admission laboratory work was significant for hyponatremia, hyperkalemia, hypercalcemia, acute kidney injury, metabolic acidosis, leukocytosis, elevated erythrocyte sedimentation rate (ESR), and elevated ferritin (Table 1). The urinalysis was only significant for trace ketones. Serum lipase level, thyroid stimulating hormone, hepatitis panel, procalcitonin, lactic acid level, transferrin, and C-reactive protein (CRP) were all unremarkable.

**Figure 1:**
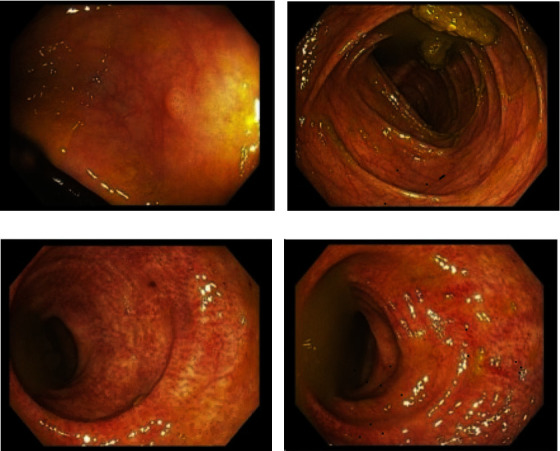
An endoscopic image showing a 5 mm polyp in the ascending colon (a) and an area of moderately congested mucosa in the transverse colon (b) and rectum (c and d).

Computed tomography (CT) scan of the abdomen and pelvis showed no intraabdominal pathology. Renal ultrasonography revealed atrophic native kidneys. The transplanted kidney was in the right lower abdomen and was normal in size and echotexture. No critical arterial or venous stenosis was observed. Owing to concerns for medication-induced colitis, we switched the patient's mycophenolate to azathioprine, but the diarrhea persisted. Infectious stool workup, including *Clostridium difficile*, ova and parasites, *Cryptosporidium*, *Salmonella*, *Shigella*, and *Giardia lamblia*, was negative. Inflammatory bowel disease workup also came back negative. Esophagogastroduodenoscopy (EGD) revealed a small hiatal hernia, bilious gastric fluid, and erythema in the gastric body and antrum. Colonoscopy demonstrated an area of moderately congested rectal mucosa and a 5 mm sessile polyp in the ascending colon (Figure 1). Random biopsies from the esophagus, stomach, colon, and rectum were obtained for histological examination, and the polyp was removed using cold biopsy forceps. The pathology results showed esophagitis, gastritis, and proctitis. Histopathology ruled out cytomegalovirus superinfection.

**Figure 2:**
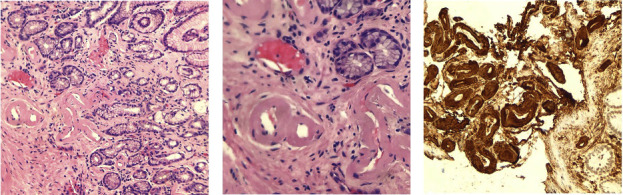
Gastric and colorectal mucosal biopsies with H&E staining (a and b) showing pinkish proteinaceous deposits consistent with amyloid deposition. (c) An electron microscopy image showing amyloid deposits in small blood vessels and smooth muscles in the gut.

While inpatient, the patient was tapered off the total parenteral nutrition and underwent a percutaneous endoscopic gastrostomy (PEG) tube placement due to severe malnutrition. With a possible diagnosis of adult-onset FMF due to recurrent episodes of serositis, fever, and abdominal pain, the patient was started on colchicine 0.3 mg, twice daily as treatment for FMF-induced gastrointestinal amyloidosis. Additional tests ruled out multiple myeloma, thyroid disease, systemic lupus erythematosus, and other rheumatological diseases (Table 1). Once the diarrhea improved, he was discharged home with plans for outpatient follow-up with a rheumatologist and an oncologist. The histopathology results of the stomach body, antrum, transverse colon, and rectum biopsies returned positive for amyloid AA (Figure 2), consistent with gastrointestinal amyloidosis. A renal biopsy ruled out organ rejection and renal amyloidosis. The patient was notified about the results, and his colchicine dose was increased to 0.6 mg twice daily, and he continued to improve.

The patient was readmitted after two months for PEG tube removal, and he was started on a regular diet with a mechanical soft consistency. Even though the patient declined a request for genetic testing to confirm an FMF diagnosis, he met the full major Tel-Hashomer diagnostic criteria for FMF. That is, he experienced recurrent episodes of serositis with fever, had biopsy-confirmed SAA amyloidosis without a predisposing disease, and improved rapidly with colchicine therapy. We diagnosed the patient with FMF and FMF-induced gastrointestinal amyloidosis. The patient continues to follow up with our continuity clinic, and he remains on colchicine for FMF prophylaxis. No one in the patient's immediate family (grandparents, parents, siblings, and children) has similar symptoms or a documented diagnosis of FMF.

## 3. Discussion

FMF is the most frequent periodic syndrome and is characterized by recurrent attacks of polyserositis and fever [2, 4]. FMF attacks are acute [2] and have no identifiable triggers. Fever is the most prevalent symptom among patients with FMF [1]. FMF attacks may manifest as pleurisy, erysipelas-like rash, arthritis, recurrent abdominal pain, or an acute scrotum [3]. Patients with M694V homozygosity have a severe form of FMF and an earlier onset [2]. These patients experience more frequent flares and require higher doses of colchicine as a prophylaxis. They also have a higher incidence of complications, such as gastrointestinal (GI) amyloidosis. Amyloidosis is the extracellular deposition of misfolded fibrillar proteins in tissues, disrupting tissue anatomy and function [4, 7–9]. GI amyloidosis often presents as recurrent abdominal pain, nausea, vomiting, weight loss, diarrhea, constipation, bleeding, fecal incontinence, dysphasia [7, 10, 11], or hepatomegaly [12]. Gut involvement occurs in 3–8% of all cases of secondary amyloidosis cases [5].

Intractable diarrhea is the most common symptom in GI amyloidosis cases [5, 11]. Typically postprandial, the diarrhea is often watery. The mechanism is not fully understood but is postulated due to autonomic neuropathy and intestinal inflammation [11]. Our patient presented with intractable watery diarrhea for over six months, which was found to be due to GI amyloidosis. SAA deposition in the stomach causes early satiety and reduced appetite leading to unintentional weight loss. Chronic diarrhea, malabsorption, nausea, and vomiting can also contribute to the cachexia [11]. Rarely, secondary amyloidosis can manifest as lower GI bleeding, mainly in the setting of tissue ischemia. Parts of the GI tract without a collateral blood supply are the most affected [5]. Hashmi et al. [5] presented an interesting case of rectal proctitis as a unique complication of secondary amyloidosis. Amyloid deposition predisposes patients to intestinal ischemia, which can cause mucosal ulcerations and vascular fragility [5]. Kim et al. [13] reported a rare case of massive hematochezia in the setting of intestinal amyloidosis that was refractory to multiple arterial embolizations. Rectal involvement is very rare owing to its dual blood supply [5]. Amyloid deposits in the esophageal smooth muscle and autonomic plexus often leads to delayed gastric emptying in patients with FMF. Saglam et al. [10] reported a rare case of delayed gastric emptying in a patient with FMF that improved with erythromycin treatment [10]. Very rarely, amyloid proteins can be found in the liver, resulting in hepatomegaly and even portal hypertension [12]. Liver transplantation has been floated as a therapeutic option in advanced cases [12].

In GI amyloidosis, routine imaging studies such as a CT scan of the abdomen may be unremarkable or show nonspecific findings such as bowel wall thickening, narrowing of the intestinal lumen, or loss of colonic haustrations [11, 12]. Upper endoscopy with biopsy of the stomach or duodenum has a higher yield of GI amyloidosis [11]. Endoscopic ultrasound, push enteroscopy, and colonoscopy have been used in some cases. Congo red staining with apple-green birefringence under polarized light is the standard modality for amyloid diagnosis. Amyloid typing is essential for identifying the amyloid subtypes and choosing appropriate treatments [11]. GI amyloidosis can be misdiagnosed because of its rarity and diverse clinical presentation.

Amyloidosis treatment entails identifying the etiology and managing the symptoms [9]. With 92% efficacy, colchicine remains the first-line treatment for FMF attacks and FMF prophylaxis [4]. Colchicine prophylaxis prevents amyloid deposition in tissues and the resulting organopathy [2, 4]. Colchicine intolerance may be due to toxicity, side effects, or resistance [14, 15]. Isolated cases of FMF-induced gastrointestinal amyloidosis refractory to colchicine therapy have been reported in the literature. Monoclonal antibodies targeting IL-1 and IL-6 receptors are a promising alternative for these patients [15]. Anakinra, a recombinant IL-1 receptor antagonist, reduces the frequency of FMF attacks and improves renal function in patient with renal amyloidosis [15]. Despite limited sample sizes, Canakinumab, another IL-1 receptor antagonist, has shown almost equal efficacy as Anakinra in clinical trials [15]. It is an option for patients who are intolerant to Anakinra due to its side effect profile or lack of therapeutic benefit [14, 15]. Tocilizumab, an IL-6 antibody, works by suppressing the pyrin inflammasome complex, reducing the frequency of FMF attacks and the incidence of complications such as gastrointestinal amyloidosis [2, 4]. Hamanoue et al. [2] reported a case of secondary amyloidosis refractory to colchicine therapy, which was successfully treated with tocilizumab. Similarly, Aikawa et al. [4] reported an atypical case of FMF with persistent arthralgia and intractable diarrhea that was resolved with tocilizumab therapy. Tocilizumab can be considered a second or third line for patients with FMF or FMF-associated amyloidosis refractory to colchicine.

## 4. Conclusion

Although rare, gastrointestinal amyloidosis should be considered in the differential diagnosis for intractable diarrhea in patients with confirmed or suspected FMF. The nonspecific nature of the symptoms can delay diagnosis or lead to unnecessary surgical interventions.

## Figures and Tables

**Table 1:** The patient's admission and pertinent laboratory values compared to the reference ranges.

Variables	Values	Reference range
Sodium	129	135–145 mEq/L
Potassium	5.7	3.5–5.0 mEq/L
Calcium	10.8	8.6–10.3 mg/dL
Blood urea nitrogen	31	7–23 mg/dL
Creatinine	1.34	0.60–1.24 mg/dL
Uric acid	6.3	2.3–7.6
Lipase level	20	8–57 U/L
Serum bicarbonate	18	21–31 mg/dL
Free T4	0.98	0.61–1.12 ng/dL
TSH	6.525	0.450–5.330 mcIntlUnit/mL
Hemoglobin	8.3	13.5–17.5 g/dL
White blood cells	12.1 × 10^3^/mm^3^	4.5 × 10^3^/mm^3^–11.0 × 10^3^/mm^3^
ESR	31	0–10 mm/hr
CRP	4.1	≤9.9
Ferritin	1,300.0	16.4–294.0 ng/mL
B12	602	211–911 pg/mL
Folate	4.0	≥4.1 ng/mL
Transferrin	166	201–355 mg/dL
Cortisol	6.7	Normal
Procalcitonin	0.14	Normal
Lactic acid	1.4	
Tacrolimus level	8.6	2.0–20.0
ANA with reflex		Negative
MPO-ANCA and PR3-ANCA		Not detected
Hepatitis B and C profile		Normal
Serum protein electrophoresis		Normal
C diff toxin		Negative
C diff antigen		Negative
SAA		Positive on biopsy

## Data Availability

All data used to support the findings of this case report are available as part of the article and references.
